# Phylogeography and taxonomic status of the Formica picea complex (Hymenoptera: Formicidae)

**DOI:** 10.18699/vjgb-26-31

**Published:** 2026-04

**Authors:** Z.A. Zhigulskaya, S.V. Shekhovtsov, S.V. Chesnokova, A.P. Burnasheva, A.A. Gurina, R.Yu. Dudko, T.V. Poluboyarova, S.V. Reshetnikov, Yu.N. Sundukov, D.I. Berman

**Affiliations:** Institute of Biological Problems of the North of the Far Eastern Branch of the Russian Academy of Sciences, Magadan, Russia; Institute of Biological Problems of the North of the Far Eastern Branch of the Russian Academy of Sciences, Magadan, Russia Institute of Cytology and Genetics of the Siberian Branch of the Russian Academy of Sciences, Novosibisk, Russia; Institute of Systematics and Ecology of Animals of the Siberian Branch of the Russian Academy of Sciences, Novosibirsk, Russia; Institute of Biological Problems of Cryolithozone of the Siberian Branch of the Russian Academy of Sciences, Yakutsk, Russia; Institute of Systematics and Ecology of Animals of the Siberian Branch of the Russian Academy of Sciences, Novosibirsk, Russia; Institute of Systematics and Ecology of Animals of the Siberian Branch of the Russian Academy of Sciences, Novosibirsk, Russia; Institute of Cytology and Genetics of the Siberian Branch of the Russian Academy of Sciences, Novosibisk, Russia; Institute of Systematics and Ecology of Animals of the Siberian Branch of the Russian Academy of Sciences, Novosibirsk, Russia; Federal Scientific Center of the East Asia Terrestrial Biodiversity, Far Eastern Branch of the Russian Academy of Sciences, Vladivostok, Russia; Institute of Biological Problems of the North of the Far Eastern Branch of the Russian Academy of Sciences, Magadan, Russia

**Keywords:** Formica picea, Formica candida, cryptic species, Northern Eurasia, Siberia, Formica picea, Formica candida, виды-двойники, Северная Евразия, Сибирь

## Abstract

The black bog ant Formica picea complex is widespread from the Atlantic to the Pacific coasts of Eurasia. This complex was earlier believed to consist of one or two species (F. picea and F. candida). However, molecular analysis suggested that it includes three cryptic species. One is F. picea from Europe, another, F. candida, is currently known exclusively from Kyrgyzstan, while the third one, temporarily designated here as Formica sp., inhabits the easternmost part of Eurasia from China to Kamchatka. It is unknown how F. picea and Formica sp. are distributed in Siberia and whether their ranges intersect. Here we studied a sample of this complex from Siberia using mtDNA and found that their ranges overlap. The distribution of Formica sp. extends from the south of West Siberia, including Altai, to China, and the Russian Far East. No phylogeographic structure was detected, suggesting their recent dispersal from a single source. F. picea was found as far as East Siberia, but was relatively rare. While the European and West Siberian populations were genetically closely related, the specimens from Zabaykalsky Krai differed, suggesting a putative East Siberian refugium. We also determined that ecologically F. picea inhabits peat bogs in lowland areas and grassy communities above the tree line in the European mountains; in Altai, it is found in mountain steppes, while in Transbaikalia, in waterlogged areas along riverbanks. Formica sp. thrives in dry steppes and low riverbanks, but avoids bogs. Thus, F. picea and Formica sp. differ genetically, and have different distribution ranges, as well as habitat preferences. This supports the opinion that Formica sp. should be recognized as a distinct species.

## Abstract (ru)

Комплекс Formica picea, или черный болотный муравей, широко распространен по всему северу Евразии, от Испании и Ирландии до побережья Тихого океана и Тибета, примерно между 35 и 66.5° северной широты. Ранее считалось, что он состоит из одного или двух видов (F. picea и F. candida). Однако молекулярный анализ показал, что в него входят три криптических вида. Один из них – F. picea из Европы, другой – F. candida, известный исключительно из Киргизии, а третий, временно обозначенный как Formica sp., обитает на востоке Евразии: от Китая до Камчатки. До сих пор было неизвестно, каково распределение F. picea и Formica sp. в Сибири, пересекаются ли их ареалы. Мы изучили образцы этого комплекса из Сибири с использованием митохондриальной ДНК и обнаружили, что их ареалы перекрываются. Ареал Formica sp. охватывает юг Западной Сибири, включая Алтай, на восток – до Тихоокеанского побережья. Филогеографической структуры не обнаружено, что указывает на недавнее расселение из одного источника. Мы нашли мало образцов F. picea в Сибири, это свидетельствует, что здесь этот вид встречается редко. Самая восточная находка вида была сделана в Забайкальском крае. F. picea обитает на торфяных болотах в пределах равнин и травяных сообществах выше линии леса в европейских горах; на Алтае – в горных степях, а в Забайкалье – на влажных участках у речных берегов. Formica sp. предпочитает сухие степи и низкие берега рек, но избегает болот. Таким образом, оказалось, что F. picea и Formica sp. отличаются генетически, имеют разные ареалы и биотопические предпочтения по местообитаниям. Это свидетельствует в пользу того, что Formica sp. можно считать отдельным видом.

## Introduction

**Fig. 1.**
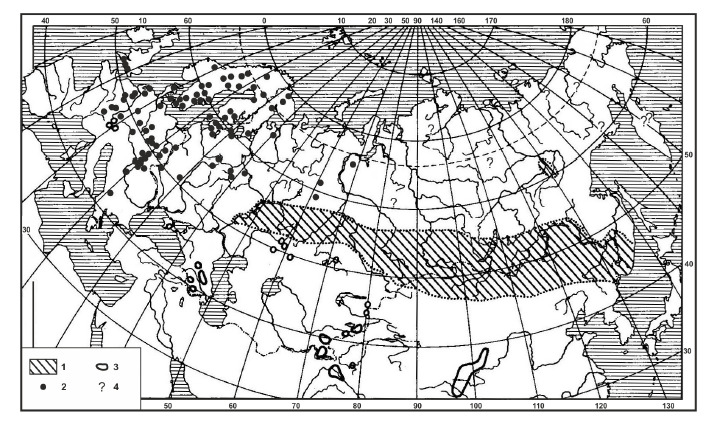
Geographical distribution and ecology of the F. picea complex. Characteristic habitats: 1 – diverse habitats; 2 – peat bogs; 3 – mountain meadows; 4 – no details provided (scheme from (Dlussky, 1967)).

The Formica picea Nylander, 1846 species complex, i. e. black bog ant, is widespread in the Palearctic, from Spain and Ireland to the Pacific coast and Tibet, and between approximately 35 and 66.5° N latitude (Ruzsky, 1905; Pleshanov, 1966; Dlussky, 1967; Dmitrienko, Petrenko, 1976; Kupyanskaya, 1990; Berman et al., 2007; Antonov, Pleshanov, 2008; Radchenko, 2016; Seifert, 2018) (Fig. 1). For a long time, F. picea was considered a single species with high geographic variation (Dlussky, 1967). Bolton (1995) proposed that the name F. candida Smith, 1878 takes precedence over F. picea. Not all specialists adopted this suggestion, leading to confusing parallel usage of both names in the literature.

Seifert (2004) suggested that F. picea and F. candida are two distinct species, based on their pubescence and certain morphometric parameters. Seifert (2004) also designated a neotype of F. candida collected from Kyrgyzstan due to the loss of the original type specimen. However, Zakharov et al. (2019) disagreed with Seifert’s conclusions, pointing out the smooth gradient of morphological variation from Europe to the Far East.

There were several molecular studies that included members of the F. picea/F. candida complex (Goropashnaya, 2003; Goropashnaya et al., 2012; Antonov, Bukin, 2016; Chen, Zhou, 2017; Schär et al., 2018). However, none of these aimed to explore the differences between the two taxa, instead simply using one or the other name. Moreover, some authors used the mitochondrial cytb gene, and others, cox1/COI, so their results could not be directly compared.

A recent study on the F. picea complex showed that it included one more species, in addition to F. picea and F. candida (Zhigulskaya et al., 2022). While F. picea turned out to be restricted to Europe, as well as to one location from Tibet, ants from all east Eurasian locations (lowland China and the Far East of Russia) were highly genetically divergent from other known Formica species. Geographic and genetic differences suggest that it should be regarded as a distinct species, although the formal description was not yet performed. Thus, this new taxon is hereafter referred to as Formica sp. There seem to be no obvious morphological distinctions for Formica sp. that could clearly differentiate it from F. picea and F. candida, supporting the observations of Zakharov and Dlussky (Zakharov et al., 2019) on high levels of intrapopulation and geographic variation for this complex.

Siberia currently remains a blank spot in this respect: it is unknown how far F. picea goes eastwards from the Urals, and how far west can Formica sp. be found. In this study we collected an extensive sample from different parts of Siberia in order to determine the ranges of both taxa, as well as to infer phylogeographic patterns. In order to search for putative ecological differences between F. picea and Formica sp., we also documented habitat preferences, which had been previously well described for European F. picea (Dlussky, 1967; Zakharov, 2015; Radchenko, 2016; Seifert, 2018; Zakharov et al., 2019).

## Materials and methods

**Fig. 2.**
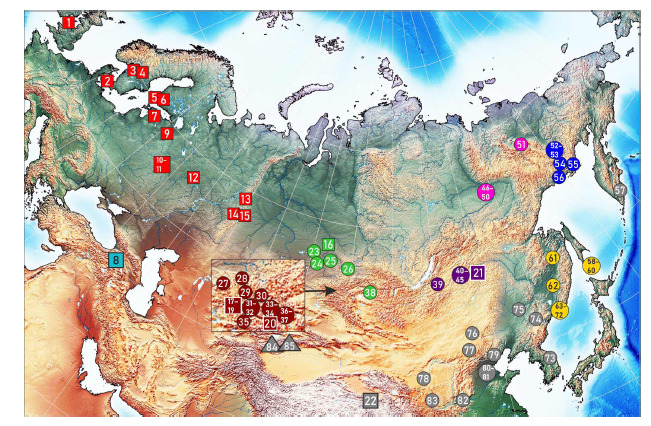
F. picea complex specimens used in this study Location numbers refer to the Table. Rectangles: F. picea; circles: Formica sp.; triangles: F. candida.

**Fig. 3.**
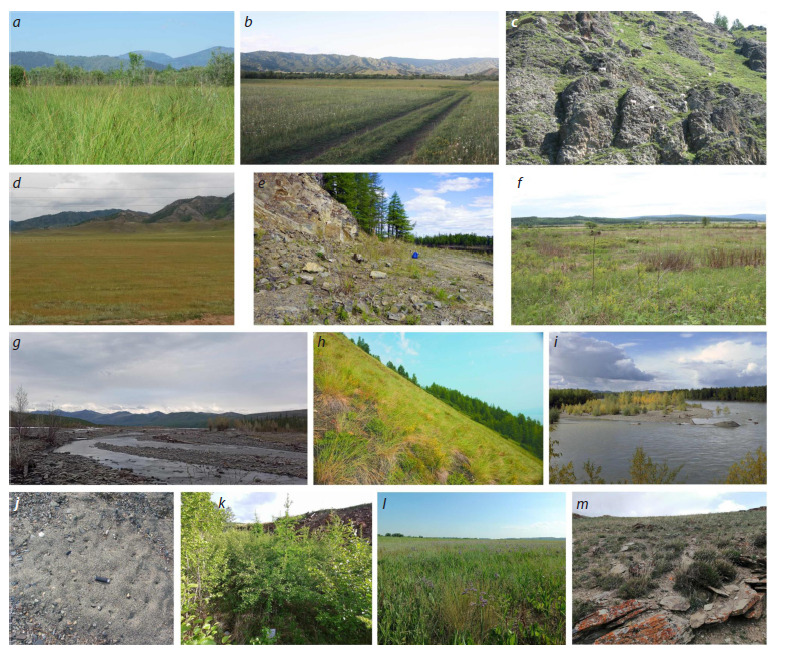
Habitats of Formica sp. and F. picea. a – Central Altai, near Abay village, lowland swamp (F. picea); b – the same area; meadows on the site of drained swamps (co-occurrence of Formica sp. and F. picea); c – the same area; stony steppes on rocky outcrops (F. picea); d – Central Altai, near Ust-Kan village, steppe (Formica sp); e – Magadan oblast, Nalednyy River (tributary of the Ola River) (Formica sp.); f – Zabaikalsky Krai, floodplain of the Talovka River (Formica sp.); g – Sakha Republic (Yakutia), Momsky District, Khairdakh River (Formica sp.); h – Magadan oblast, upper reaches of the Kolyma River, steppe (Formica sp.); i – Magadan oblast, an island in the upper reaches of the Kolyma River near the eastern end of the Big Ananchag Range (Formica sp.); j – same area; nest exits of Formica sp.; k – Zabaikalsky Krai, floodplain of the Kotikha River (F. picea); i – Novosibirsk oblast, Kabinetnoye village, halophytic meadow (Formica sp.); m – Southeast Altai, near Kosh-Agach village, Tabozhok town, stony steppe (F. picea). For more details, see the Table.

We surveyed a wide range of habitat types, from steppes to bogs, in both lowland and mountainous landscapes of Siberia and the Russian Far East (see the Table, Figs. 2, 3). Ants were collected into 96 % ethanol in a series of 6–10 specimens from nests, as well as from forage plants. The collection material is stored in the Institute of Systematics and Ecology of Animals, Siberian Branch of the Russian Academy of Sciences (Novosibirsk, Russia).

**Table 1.**
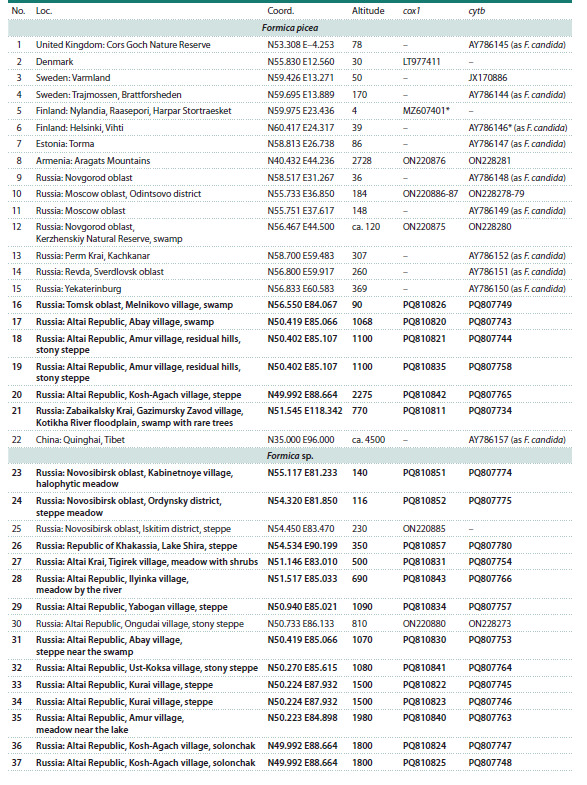
Representatives of the F. picea complex with available cox1/cytb sequences Note. Loc. – location numbers referring to Fig. 2; altitude provided in m above sea level. Asterisks denote type of material/locations. Specimens sequenced in this study are indicated in boldface.

**Table 1continued.**
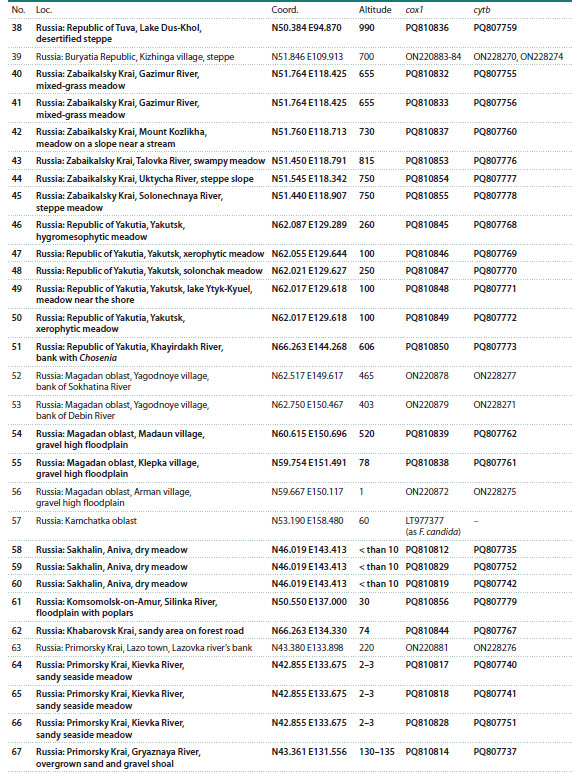
Table 1continued

**Table 1end.**
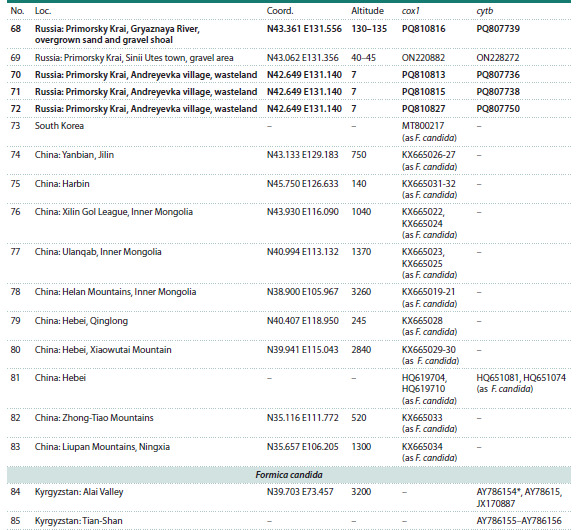
Table 1end

Ants of the F. picea complex were taken for genetic analysis from 85 locations (Fig. 2): for 47, obtained by us in this study, for the rest, they were taken from our previous study (Zhigulskaya et al., 2022) and from GenBank.

DNA was extracted from individual worker ants preserved in ethanol using commercial silica columns (BioSilica, Novosibirsk, Russia), following the method outlined in (Shekhovtsov et al., 2020). We amplified a segment of the mitochondrial cytochrome c oxidase subunit 1 (cox1) gene with the universal primers LCO1490m (5′-TACTC-AACAA-ATCACAAAGA- TATTG-G-3′; modified from (Folmer et al., 1994)) and HCO2198 (5′-TAAAC-TTCAG-GGTGA-CCAAAAAATC- A-3′) (Folmer et al., 1994). Additionally, we amplified a portion of the cytochrome b (cytb) gene using primers Fcbl- F (5′-ACCCT-CACCT-GTAAA-TATTT-CTT-3′) and Fcbl-R (5′-GGAAT-AGATC-GTAAA-ATTGC-AT-3′) (Zhigulskaya et al., 2022). PCR was conducted with the Biomaster HS-Taq PCR Mix (Biolabmix, Novosibirsk, Russia).

The DNA fragments obtained were visualized through agarose gel electrophoresis. Unincorporated primers and nucleotide phosphates were eliminated using shrimp alkaline phosphatase and Escherichia coli exonuclease I mixture (New England Biolabs, Ipswich, MA, USA). Sanger sequencing was conducted on a 3130xl DNA Analyzer (Applied Biosystems, Framingham, MA, USA) at the SB RAS Genomics Core Facility (ICBFM SB RAS, Novosibirsk, Russia) using both forward and reverse primers. The resulting sequences were submitted to GenBank with accession numbers PQ807734–PQ807780 (cox1) and PQ810811–PQ810857 (cytb). Sequences from other researchers were also incorporated into this study (refer to the Table for GenBank accessions). This dataset includes the cytb sequence for the F. candida neotype (AY786154). The haplotype network was constructed using the median joining algorithm in Pop ART v. 1.7 (Leigh, Bryant, 2015).

## Results

**Fig. 4.**
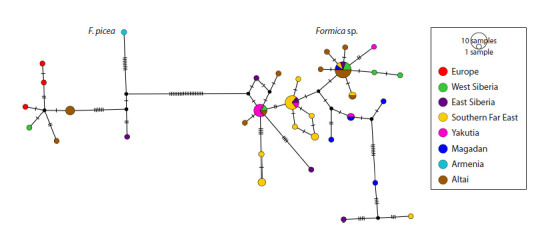
Haplotype network for the combined cox1+cytb sequences. Dashes represent the number of nucleotide substitutions.

Out of the 47 ant specimens sequenced in this study for cox1 and cytb, 6 belonged to F. picea, and 41, to Formica sp. We did not detect haplotypes of F. candida (Seifert, 2004) in any of the sampling points, despite the extensive survey area. F. picea and Formica sp. significantly differed at the DNA level (Fig. 4).

For F. picea, a notable geographic split was evident: samples from Europe and West Siberia formed a tight cluster, while samples from Armenia and Transbaikalia diverged from them (Fig. 4). For Formica sp., the larger sample size resulted in a more informative network. However, no regions with a specific set of haplotypes were identified. Genetic diversity was substantial, especially for three samples (from different regions) that differed markedly from other haplotypes.

F. picea was found in five locations east of the Urals: in the Altai mountains, in the lowlands of West Siberia, as well as in East Siberia. The West Siberian location (Melnikovo, Tomsk oblast) was in a fen with a tall birch. The nest (with no external structures) was located in a sedge hummock with a fern; nest entry was in moss

In the Altai mountains, we found nests of F. picea: three in Central Altai near the Abay (locations No. 17 in the Table and Fig. 2) and Amur (locations No. 18–19 in the Table and Fig. 2) villages (ca. 1100 m a. s. l.), and one in the Southeast Altai (locations No. 20 in the Table and Fig. 2), near the Kosh- Agach village (2275 m a. s. l.). Near the Abay village, a single nest was detected in a damp area on the edge of a bog, where it transitioned into a steppe meadow, 150–200 m away from a small lake. The nest had a ground part in the form of a small dome made of thin blades of grass, no more than 10 cm high and about 15 cm in diameter. Two other nests were found on southern steppe slopes, with no external structures and exits located under the rocks.

In East Siberia, F. picea was detected in Zabaykalsky Krai, east of the Gazimursky Zavod town (locations No. 21 in the Table and Fig. 2), at 770 m a. s. l., within the forest belt. The nest was found on a swampy floodplain of the Kotikha River (a third-order tributary of the Gazimur River), with saplings of alder, birch, and larch. The nest, without visible external structures, was located under a layer of litter composed of fallen leaves and dry grass.

Specimens of Formica sp. were detected in 61 locations (Fig. 2). Their habitats included floodplain (including longterm and deeply flooded sandy-pebble spits) and non-floodplain terraces of different heights on rivers of various sizes, meadows of diverse composition and projective cover, sparse forests, steppes (from mixed grass to desert), foothills and highlands of Altai. This ant was found to avoid forests with closed canopies, as well as wetlands.

## Discussion

**Genetic patterns**

As in the previous study (Zhigulskaya et al., 2022), F. picea and Formica sp. were significantly different on mtDNA levels, with no intermediate haplotypes found (Fig. 4). Expectedly, we found no haplotypes of F. candida sensu Seifert (2004), which is probably limited to certain regions of Kyrgyzstan.

Throughout the distribution of Formica sp., from the Altai mountains to the Pacific coast and eastern China, there is a pretty high genetic variation, but with no obvious genetic structure (Fig. 4). This probably indicates recent colonization of this region without a bottleneck event and high dispersal abilities of the species. Unfortunately, there are no cox1 data on Formica sp. from China, a region with a milder climate that could serve as a refugium/ancestral region of Formica sp.

Noteworthy, three haplotypes differed by 8–15 nucleotide substitutions from the main cluster (Fig. 4). However, all three were from different and geographically remote regions. This might imply that the history of the species is complex, with high dispersal rate and multiple colonization waves.

Although the data on the cox1 gene in F. picea remain limited, several interesting insights can be gleaned from the haplotype network (Fig. 4). Most haplotypes form a tight cluster, indicating close genetic relationships. This cluster comprises specimens from the Altai and West Siberia, suggesting recent (likely Holocene) dispersal into these regions. The Armenian haplotype is distantly related to this group, as expected given the geographic distance and mountain barriers. Notably, the East Siberian haplotype is also distinct from the European cluster. Intriguingly, this pattern may indicate that East Siberian populations of F. picea belong to a different genetic lineage relative to the European/West Siberian group, and that an East Siberian refugium for the species may have existed. Of course, drawing firm conclusions based on a single specimen would be premature; thus, we intend to examine additional specimens from the region in future studies.

**Geographic distribution**

There are a lot of studies on the distribution and habitat preferences of the F. picea/F. candida complex. In various parts of its range, these ants are known to inhabit diverse habitats: fens, meadows, steppes, as well as sparse forests on plains, floodplains, and in highlands. In the south taiga zone of Siberia, black bog ants are found in swamps, in wet mixed forests, in pine woods (Omelchenko, Zhigulskaya, 1998; Dlussky, 2001; Gridina, 2003). They are ubiquitous in Central Yakutia and the Baikal Region, from boggy larch forests, dry pine-larch forests, and steppe-like and steppe biotopes to bare rock formations, with nest densities reaching up to 30 nests per 100 square meters (Pleshanov, 1966; Dmitrienko, Petrenko, 1976).

However, they are particularly ecologically versatile and abundant in the mountain-steppe landscapes of Tuva, Southeast and Central Altai, as well as in the steppes of Transbaikalia, where they dominate almost all habitats, from solonetzes and solonchaks to dry steppes on sandy and stony soils (Zhigulskaya, 1968, 2009, 2011; Chesnokova, Omelchenko, 2011).Since the black bog ant turned out to include several cryptic taxa, this raises a question if they have different habitat preferences. Here we obtained certain data on this point, which are, of course, far from exhaustive. For F. picea, we currently know that it is found from West Europe to West Siberia. We also have single findings from Transbaikalia (this study) and Tibet (AY786157), implying the presence of this taxon between Siberia and Tibet. F. picea appears to be rare in Asia, since we found only six locations of this species compared to 41 for Formica sp. Of these, only two were not from the mountains: a location in Tomsk oblast and one from mid-altitude highlands

In Northeast Asia, the complex is associated primarily with river valleys; high abundance is observed in sparse Populus and Chosenia groves and on gravel-sandy spits in floodplains. Much less frequently, and only as isolated nests, Formica sp. can be found in mesoxeromorphic sparse forests (in Yakutia, specifically in pine forests), in steppe habitats, or southern slopes with sphagnum-moss bogs (Berman et al., 2007). 

Since the black bog ant turned out to include several cryptic taxa, this raises a question if they have different habitat preferences. Here we obtained certain data on this point, which are, of course, far from exhaustive. For F. picea, we currently know that it is found from West Europe to West Siberia. We also have single findings from Transbaikalia (this study) and Tibet (AY786157), implying the presence of this taxon between Siberia and Tibet. F. picea appears to be rare in Asia, since we found only six locations of this species compared to 41 for Formica sp. Of these, only two were not from the mountains: a location in Tomsk oblast and one from mid-altitude highlands of Transbaikalia. Based on our findings, we can suggest that F. picea appears to be associated with fens, but not peatlands. The latter is probably due to the fact that peatlands in Siberia are still insufficiently studied by myrmecologists.

Formica sp., on the other hand, appears to be found even more widely than previously thought (Zhigulskaya et al., 2022). In this study it was recorded across a vast area, from West Siberia to the Pacific Ocean, and from 66.5°N in Yakutia (Khayyrdakh River) roughly to 35°N in the southern part of the Korean Peninsula (Fig. 2), as well as from sea level up to approximately 3500 m a. s. l., across a broad range of habitats (except for forests with closed canopy). Furthermore, unlike F. picea, Formica sp. has not been found in bogs in Europe, Siberia, or Altai. Conversely, only Formica sp. is known from river floodplains, and from these areas, it can spread to xeromorphic slopes and sections of low floodplain terraces.

The versatility of Formica sp. to moisture is striking: it can colonize both long-term flooded habitats on floodplains (that is, for about a month of the 4.5-month active period in the Subarctic) and dry zonal steppes. Notably, in the Altai Mountains, Formica sp. nests were found in both types of habitats

In this study, we found that the distributions of Formica sp. and F. picea significantly overlap, probably from the Urals to Transbaikalia (Fig. 2), and we expect this overlap to expand when new data on bog ants are obtained. However, the ecological preferences of these two taxa seem to differ, which can be prospectively formulated as follows: Formica sp. avoids fens, while F. picea does not like low floodplains.

In this connection, the cases of syntopic occurrences of both taxa in the Central Altai are of particular note. Here, these two nests were found a few dozens of meters apart on the floodplain of the Abay River. The nest of F. picea was at the edge of a wet swampy depression about 1.5 m deep, while that of Formica sp. was on a dry steppe meadow that was formed in a dried fen. In the same location, about 3 km apart, we found a F. picea nest on a southern slope of the residual hills with rock outcrops, occupied by steppes

We can suggest that in the Altai, F. picea inhabits meadows, and can move into both wet habitats and moderately dry steppes on southern slopes, as well as mountain steppes above the forest edge. However, it is unclear why this species can use both very wet and very dry habitats (Bönner, 1915; Skwarra, 1929; Stitz et al., 1939; Seifert, 2004, 2018).

To this, we can put forward the hypothesis that the generalist F. picea has to constrict itself to the habitats where it can compete with other ant species (Dlussky, 1967). “Apparently, while spreading westward, F. picea (a eurytopic forest-steppe species of Mongolian origin) was displaced by species of the F. rufibarbis group into bogs. In these bogs, the F. rufibarbis species are virtually absent, whereas F. picea can survive, owing to its eurytopy and plasticity in nest construction. In the mountains, this species inhabits higher altitudes than other species of the F. rufibarbis group (except for F. subpilosa pamirica and litoralis in Central Asia, which exclusively occupy river thalwegs, rocky screes, and sands). F. picea cannot compete with F. fusca and its closely related species, as it is virtually absent in forests; instead, it is associated with more or less open habitats (meadows, steppified areas, sparse forests, river floodplains, and bogs)” (translated from: Dlussky, 1967, p. 63).

If this idea is correct, Formica sp. outcompetes F. picea on the vast floodplains and xeromorphic habitats of Asian Russia. So, F. picea is outcompeted both in Siberia and in Europe by different local ant species.

## Conclusion

We can summarize the following important points inferred from our study: only F. picea is found in Europe, whereas both F. picea and Formica sp. are found in North Asia. We failed to detect F. candida on this vast territory, and we can surmise that in the current understanding proposed by Seifert (2004), it is a Central Asian taxon. In the south of West and East Siberia, the ranges of F. picea and Formica sp. overlap, but they have different ecological preferences. F. picea is probably constrained to fens on the plains, and to subalpine meadows in the mountains. Formica sp. is broadly tolerant to humidity but is not found in fens. The cases of syntopic distribution of these ants, which we described in Altai, are apparently associated with transient successional processes. The relative rarity of F. picea in our collections possibly reflects the insufficient study of raised bogs in Asia, the typical habitats of the species in Europe. Differences in the distribution and habitat preference of F. picea and Formica sp. are probably the result of competition, according to G.M. Dlussky (1967).

F. picea and Formica sp. differ genetically and have different distribution ranges, as well as habitat preferences. This supports the opinion that Formica sp. should be recognized as a distinct species

## Conflict of interest

The authors declare no conflict of interest.
